# Effect of individualized treatment strategy on postoperative nausea and vomiting in gynaecological laparoscopic surgery: a double-blind, randomized, controlled trial

**DOI:** 10.1186/s12871-022-01809-z

**Published:** 2022-08-19

**Authors:** Wenjing Ma, Yupeng Qi, Can Liu, Mingfang Wang, Yun Zhang, Weidong Yao

**Affiliations:** 1 Department of Anesthesiology, First Affiliated Hospital of Wannan Medical College, No. 2 Zheshan Street, Wuhu, 241000 Anhui China; 2 Department of Critical Care Medicine, First Affiliated Hospital of Wannan Medical College, No.2 Zheshan Street, Wuhu, 241000 Anhui China

**Keywords:** Postoperative nausea and vomiting, Gynaecological laparoscopic surgery, Individualized treatment strategy

## Abstract

**Background:**

Postoperative nausea and vomiting (PONV) have always been a concern of clinicians and may increase medical costs for patients. Consensus guidelines recommend using multiple antiemetics with different mechanisms as prophylaxis in patients at high risk of PONV. Individualized risk scores for nausea and vomiting and individualized treatment strategies are feasible. This study evaluated the effect of individualized treatment strategies on postoperative nausea and vomiting after laparoscopic gynaecological operations.

**Methods:**

This was a double-blind, randomized, controlled trial. A total of 119 adult patients who underwent gynaecological laparoscopic surgery under general anaesthesia were randomly divided into an individualized treatment group or a control group, with the individualized treatment group receiving individualized prevention according to a preoperative risk score of nausea and vomiting and the control group receiving no individualized prevention. Vomiting, retching, nausea, and use of rescue medication were all recorded for 24 h after the operation. The primary outcome variable was complete response, defined as no emesis or the use of rescue medication 24 h postoperatively.

**Results:**

The complete response rate was higher in the individualized treatment group (56.7%) than in the control group (23.7%) (95% CI, 0.01–0.27; *P* < 0.001). The incidences of emesis (18.3% *vs.* 44.1%, *P* = 0.002) were significantly lower in the individualized treatment group than in the control group. There were no differences in any nausea (26.7% *vs.* 33.9%, *P* = 0.391) or rescue medication use (6.7% *vs.* 8.5%, *P* = 0.743). Adverse events and laboratory and electrocardiogram abnormalities occurred no more frequently in the individualized treatment group than in the control group.

**Conclusion:**

In conclusion, this single-centre, double-blind, randomized study suggests that an individualized PONV prophylactic treatment strategy based on the number of PONV risk factors could be a safe and effective regimen to reduce the incidence of PONV in adult patients undergoing laparoscopic gynaecological surgery.

## Introduction

Postoperative nausea and vomiting (PONV) is one of the most frequent common complications of surgical procedures, occurring in approximately 30% of all surgical patients and more than 70% of certain high-risk patients [1–4]. The study by Kawano [5] showed that the incidence of nausea or vomiting is approximately 72% in patients undergoing laparoscopic gynaecological surgery.

Patient-specific risk factors for PONV in adults include female sex, prior history of postoperative nausea and vomiting or motion sickness, nonsmoking, and the use of postoperative opioid analgesia [6–8]. The simplified risk score proposed by Apfel was widely used to predict patients’ risk for PONV. Consensus guidelines recommend using multiple antiemetics, including serotonin 5-HT_3_, dopamine D_2_ and histamine H_1_ antagonists, and corticosteroids as prophylaxis in patients at high risk of PONV [9–12]. The latest PONV guidelines suggest the use of multimodal prophylaxis in patients with one or more risk factors [13]. It has been shown that antiemetics of different mechanisms provide additive benefits, each reducing the relative risk of PONV by approximately 25% [14].

Therefore, based on the above background, we proposed a simple and feasible treatment strategy for PONV based on the preoperative risk factor scores of nausea and vomiting to guide anaesthesiologists in the use of antiemetic drugs. We conducted this study to test the hypothesis that the individualized treatment strategy for nausea and vomiting when used can prevent postoperative nausea and vomiting in gynaecological laparoscopic surgery patients.

## Methods

### Study design

This prospective, double-blind, randomized, controlled trial was performed between July 2021 and February 2022 at the First Affiliated Hospital of Wannan Medical College, Wuhu, China. The study protocol was approved by the local Medical Ethics Committee (No. 2021–06). All of the patients were informed about the study and signed the informed consent form. The study was conducted following the Declaration of Helsinki. The trial was registered on Chictr.org before initiation on 20/06/2021, registration number ChiCTR2100047571 (http://www.chictr.org.cn/edit.aspx?pid=128533&htm=4).

### Patient population

Eligible patients were 18–65 years of age; underwent elective laparoscopic gynaecological surgery under intravenous general anaesthesia, expected to last at least an hour; were classified as ASA I-II; were excluded if they had a known gastrointestinal disease; had taken antiemetic medication within 24 h before surgery; and were allergic to drugs.

### Study treatments

The patients were randomly assigned to 1 of the 2 following groups according to the number (from 1–2) written inside the closed envelope and given the relevant medication. A computer-generated list of random numbers was used. Before surgery, the patients were evaluated according to Apfel risk factors for PONV: female sex, previous history of postoperative nausea and vomiting or motion sickness, being a nonsmoker, and expected use of postoperative opioids for analgesia [15]. In the individualized treatment group, patients were administered according to the number of risk factors for nausea and vomiting that existed before surgery. For patients with 1 risk factor, dexamethasone was given to them intravenously before the induction of anaesthesia. Patients with 2 risk factors were given two antiemetic medications, dexamethasone plus ondansetron or granisetron intravenously before abdominal closure. Patients with more than 3 risk factors were given three antiemetic medications, such as dexamethasone plus ondansetron or granisetron plus scopolamine. The control group was given one antiemetic drug.

All operations were performed by the same team of surgeons. Anaesthesia providers were not involved in collecting the outcome data. All of the drugs used in this study were prepared for administration by the same researcher who was not involved in the perioperative treatment of the patients. The medications and the syringes were similar in appearance with respect to colour and viscosity. Additionally, the anaesthesiologist and the surgeon were unable to see the composition of the solution injected into the operating theatre. The overall blinding process was implemented until all data had been entered into the database and their accuracy confirmed. Patients and all personnel involved in the trial conduct were blinded to the treatment assignment. Rescue antiemetic medication was to be given postoperatively to any patient with nausea or emesis, with the choice of agents being determined by the gynaecologist.

### Perioperative management

Anaesthesia induction was achieved by using midazolam (0.05 mg/kg), sufentanil (0.3 μg/kg) and etomidate (0.3 mg/kg). Neuromuscular blockade was achieved by using rocuronium bromide (0.6 mg/kg IV), and endotracheal intubation was performed by inserting an orotracheal tube after sufficient muscular relaxation had been achieved. Anaesthesia was maintained with 0.1–0.2 μg/kg/min remifentanil intravenous (IV) infusion and 4–8 mg/kg/h propofol intravenous (IV) infusion. Rocuronium was statically administered intermittently to maintain muscle relaxation. After the completion of the surgery, patients with sufficient spontaneous respiration were extubated. The patients were then transferred to the postoperative anaesthesia care unit where they were monitored. Subsequently, the patients were transferred to a gynaecology clinic, where they were followed for over 48 h.

### Evaluations

The episodes of emesis (vomiting/retching), nausea, and rescue medication during the 24 h after the operation were recorded. Nausea was also assessed by direct questioning at 0–6, 6–12 and 12–24 h after the operation. The severity of nausea was evaluated on a PONV grading standard, with degree I representing no nausea or vomiting, degree II representing one or two episodes of nausea and vomiting, degree III representing three or four episodes of nausea and vomiting, and degree IV representing at least five episodes of nausea and vomiting. Complete response was defined as no emesis or the use of rescue medication within 24 h postoperatively. All adverse events were recorded from the administration of the study through discharge. Delayed recovery was defined as not being awake 90 min after anaesthesia. Arrhythmia is defined as QTc prolongation. The incidence of postoperative infection and hyperglycaemia (> 6.0 mmol/L) was recorded.

The primary outcome was no emesis or the use of rescue medication 24 h postoperatively. Secondary outcomes included the severity of nausea and vomiting, the incidence of vomiting or nausea, and adverse events.

### Statistical analysis

Based on the difference of 18% in the complete response rate of PONV, we determined that 102 patients in each group would be necessary to detect an 18% PONV with a power of 90% and a type I error of 0.05. To account for a potential 10% dropout rate, we enrolled 224 patients.

Pearson’s chi-square test was used to assess the difference between two groups.

Statistical significance was calculated using log-rank tests. The severity of nausea was analysed using a Mann–Whitney test. Where appropriate, hypothesis testing was two-tailed with significance interpreted as *P* < 0.05. SPSS software (SPSS Institute, USA), version 22, was used for all data analyses.

## Results

**Fig. 1:**
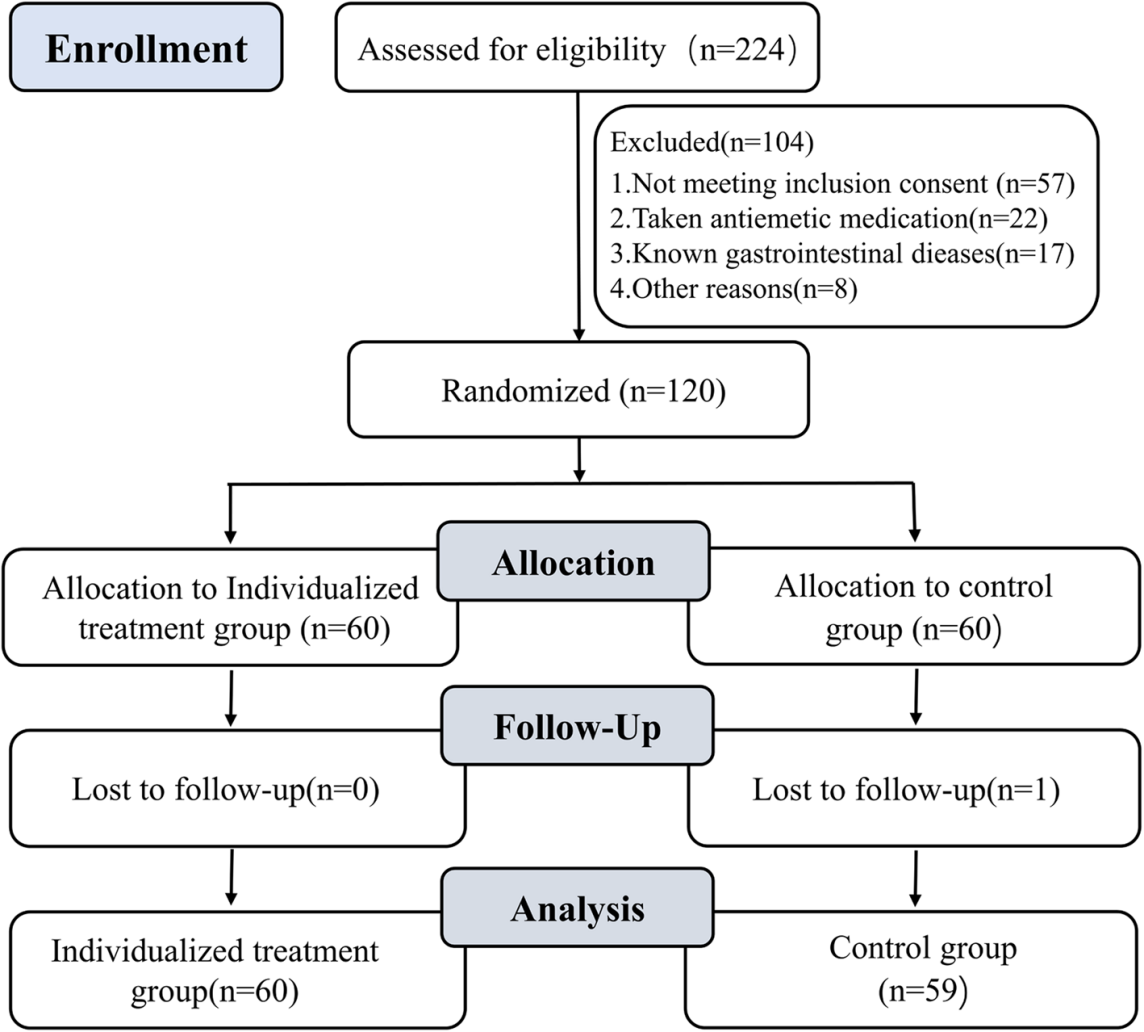
Flow diagram of the progression through the study

During the study period, 224 patients were assessed for eligibility, of whom 120 patients were enrolled in the trial. Among those 120 randomized patients, 60 patients were assigned to receive individual treatment, and 59 patients received one antiemetic drug (Fig. 1).

There was no significant difference between the two groups in age, BMI, history of PONV, history of motion sickness, nonsmoking, expected postoperative opioid use, or risk factors (Table 1).

**Table 1:** Demographic characteristics and risk factors for postoperative nausea and vomiting

	Individualized treatment group (*n* = 60)	Control group (*n* = 59)	*P*
Sex, female, n	60	59	1.000
Age, years, mean (SD)	43.3 (9.4)	43.0(9.0)	0.303
ASA classification (1/2), n	27/33	23/36	0.579
BMI, kg/m^2^, mean (SD)	22.8(2.7)	22.8(3.0)	0.782
History of PONV, n (%)	9(15.0)	8(13.6)	0.882
History of motion sickness, n (%)	21(35.0)	25(42.4)	0.409
Nonsmoking, n (%)	59(98.3)	59(100)	1.000
Expected postoperative opioid use, n (%)	43(71.7)	42(71.2)	0.954

*Abbreviations*: *SD* Standard deviation, *N* Number

Complete response occurred in 56.7% of the individualized treatment group and 23.7% of the control group (difference 33 percentage points; *P* < 0.001) (Table 2). The incidence of complete response was higher in the individualized treatment group (90.9%) than in the control therapy group (22.2%) in two risk factor patients (*P* < 0.05) (Table 2). However, the incidence of complete response in both three- and four-risk-factor patients was similar (Table 2).

**Table 2:** Complete response rates of risk factor classification

	Individualized treatment group (*n* = 60)	Control group (*n* = 59)	*P*
Risk factors			0.844
1 risk factor, n (%)	1(1.7)	1(1.7)	-
2 risk factors, n (%)	11(18.3)	9(15.3)	-
3 risk factors, n (%)	35(58.3)	32(54.2)	-
4 risk factors, n (%)	13(21.7)	17(28.8)	-
CR with all patients, n (%)	34(56.7)	14(23.7)	< 0.001
CR with 1 risk factors, n (%)	1 (100.0)	1 (100.0)	1.000
CR with 2 risk factors, n (%)	10 (90.9)	2 (22.2)	0.005
CR with 3 risk factors, n (%)	19 (54.3)	10 (31.3)	0.050
CR with 4 risk factors, n (%)	5 (38.5)	2 (11.8)	0.190

*Abbreviations*: *CR* Complete response

The severity of nausea and vomiting in degrees II, III, and IV was higher in the control group than in the individualized treatment group (Table 3).

**Table 3:** Comparison of the severity of nausea and vomiting

	Individualized treatment group (*n* = 60)	Control group (*n* = 59)	*P*
Degree I, n (%)	36(70.6)	15(29.4)	< 0.001
Degree II, n (%)	14(43.8)	18(56.3)	0.377
Degree III, n (%)	6(28.6)	15(71.4)	0.027
Degree IV, n (%)	4(26.7)	11(73.3)	0.049

Degree I: no nausea or vomiting

Degree II: one or two episodes of nausea and vomiting

Degree III: three or four episodes of nausea and vomiting

Degree IV: at least five episodes of nausea and vomiting

The incidence of emesis was significantly lower in the individualized treatment group (44.1%) than in the control therapy group (18.3%) (*P* < 0.001) (Table 4).

**Table 4:** Incidence of secondary efficacy variables in the 24-h postoperative period

	Individualized treatment group (*n* = 60)	Control group (*n* = 59)	*P*
No emesis, use of rescue medication or nausea, n (%)	36(60.0)	15(25.4)	< 0.001
Any nausea, n (%)	16(26.7)	20 (33.9)	0.391
Emesis, n (%)	11(18.3)	26(44.1)	0.002
Rescue medication, n (%)	4 (6.7)	5(8.5)	0.743

Delayed recovery and postoperative infection and hyperglycaemia showed no differences between the two groups. Electrocardiogram data also showed no clinical relevant changes.

## Discussion

The results of this study showed that compared with the patients in the control group (using one antiemetic drug), the severity and frequency of postoperative nausea and vomiting in patients receiving individualized treatment were significantly reduced in patients undergoing a gynaecological laparoscopic operation under general anaesthesia. Individualized treatment was similar to the control with respect to the overall safety profile.

Despite the use of general multimodal prophylaxis for patients with any risk factors for PONV, the incidence of nausea and vomiting remained high. The incidence of nausea and vomiting was still as high as 60% for gynaecological patients [15]. The results of this study showed that each risk factor increased the incidence of postoperative nausea and vomiting by approximately 20%, which was similar to the results of previous studies. In the control group, the incidence of nausea and vomiting was 77.8%, 68.7%, and 88.2% for those with a risk score of 2, 3, and 4, respectively. In the individualized treatment group, the incidence of nausea and vomiting was 9.1%, 45.7%, and 61.5% for groups with risk scores of 2, 3, and 4, respectively. In the individualized treatment group, grade I and II nausea and vomiting mainly occurred, while in the control group, grade III and IV nausea and vomiting mainly occurred, indicating that an individualized treatment strategy can reduce the severity of nausea and vomiting.

The secondary results of this study showed that individualized management strategies were effective in improving vomiting symptoms but not in alleviating nausea, which may be related to the primary use of 5-HT_3_ antagonists rather than dopamine receptor antagonists in this study. Studies have shown that 5-HT_3_ antagonists, such as ondansetron, have generally been found to be considerably better at preventing emesis than nausea [11]. In this study, dopamine receptor antagonists were not routinely used but only as part of a remedy for nausea and vomiting. However, dopamine antagonists, such as metoclopramide, are more effective in treating nausea than emesis [16, 17].

This study has several limitations. First, only steroid, 5-HT_3_ receptor, and anticline antagonists were used in the individualized treatment group in this study, while dopamine receptor antagonists were used only as salvage antiemetic agents, resulting in slight differences in nausea and vomiting relief between the two groups. Second, all patients in the study were female, and the results may therefore not be generalizable to male patients, although this is of limited significance because men are much less frequently at high risk of postoperative nausea and vomiting. However, the evidence that antiemetic drugs with different mechanisms of action exert an additive benefit [14]. Therefore, international guidelines recommend the use of three different types of antiemetic drugs to prevent postoperative nausea and vomiting in high-risk patients.

The study showed that the individualized treatment group did not differ from the control group in the incidence of adverse events such as chills, arrhythmias, and delayed recovery.

## Conclusions

In conclusion, this single-centre, double-blind, randomized study suggests that an individualized PONV prophylactic treatment strategy based on the number of PONV risk factors could be a safe and effective regimen to reduce the incidence of PONV in adult patients undergoing laparoscopic gynaecological surgery.

## Data Availability

The datasets used and analyzed during the current study are available from the corresponding author on reasonable request.

## Abbreviations

PONV: Postoperative nausea and vomiting
CR: Complete Response
SD: Standard deviation
