# Correction: Effect of individualized treatment strategy on postoperative nausea and vomiting in gynaecological laparoscopic surgery: a double‑blind, randomized, controlled trial

**DOI:** 10.1186/s12871-023-01984-7

**Published:** 2023-01-17

**Authors:** Wenjing Ma, Yupeng Qi, Can Liu, Mingfang Wang, Yun Zhang, Weidong Yao

**Affiliations:** 1 Department of Anesthesiology, First Affiliated Hospital of Wannan Medical College, No. 2 Zheshan Street, Wuhu, 241000 Anhui China; 2 Department of Critical Care Medicine, First Affiliated Hospital of Wannan Medical College, No.2 Zheshan Street, Wuhu, 241000 Anhui China

**Correction: BMC Anesthesiol 22, 266 (2022)**

**https://doi.org/10.1186/s12871-022-01809-z**

Following publication of this article [1], readers identified an error in Table 4 that the table title was “Incidence of secondary efficacy variables in the 24-h postoperative period”. Authors have checked that “the incidence of no emesis, use of rescue medication or nausea” should be the primary outcome, and the title of table 4 should be corrected to “Incidence of primary and secondary efficacy variables in the 24-h postoperative period”. We thank the reader for “this very meaningful comment”.
